# Alcohol-Related Neurodegeneration and Recovery: Mechanisms From Animal Models

**Published:** 2008

**Authors:** Fulton T. Crews

**Keywords:** Alcoholism, alcohol dependence, alcohol and other drug (AOD) effects and consequences, binge drinking, heavy drinking, brain, brain structure, brain function, brain atrophy, risk factors, genetic factors, environmental factors, neurons, neurodegeneration, neurogenesis, human studies, animal studies, animal models

## Abstract

Human studies have found alcoholics to have a smaller brain size than moderate drinkers; however, these studies are complicated by many uncontrollable factors, including timing and amount of alcohol use. Animal experiments, which can control many factors, have established that alcohol can cause damage to brain cells (i.e., neurons), which results in their loss of structure or function (i.e., neurodegeneration) in multiple brain regions, similar to the damage found in human alcoholics. In addition, animal studies indicate that inhibition of the creation of neurons (i.e., neurogenesis) and other brain-cell genesis contributes to alcoholic neurodegeneration. Animal studies also suggest that neurodegeneration changes cognition, contributing to alcohol use disorders. Risk factors such as adolescent age and genetic predisposition toward alcohol consumption worsen neurodegeneration. Mild impairment of executive functions similar to that found in humans occurs in animals following binge alcohol treatment. Thus, animal studies suggest that heavy alcohol use contributes to neurodegeneration and the progressive loss of control over drinking. Despite the negative consequences of heavy drinking, there is hope of recovery with abstinence, which in animal models can result in neural stem-cell proliferation and the formation of new neurons and other brain cells, indicative of brain growth.

The discovery that alcoholic humans have small brains is confounded by not knowing what the brain size was before alcoholism. Smaller human alcoholic brains could be attributed to smaller brain volume increasing risk for becoming alcoholic, alcohol-induced brain shrinkage, or both. Because mammalian brains are similar, animal studies allow investigation of the brain before, during, and after alcohol intoxication, as well as investigation of other factors that complicate understanding human disease. Animal studies continue to be used to model human responses in order to understand how to better prevent and reverse human problems. One key finding from animal studies is that high blood alcohol levels which occur with binge drinking and alcoholism can cause neurodegeneration without any nutritional or other deficiency (Crews and Nixon 2008).

Neurodegeneration is defined as the loss of structure or function of brain cells, including death of neurons and other cellular components. Alcoholic neurodegeneration is subtle, widespread, and varied but can be compared with other neurodegenerative diseases (Rosenbloom and Pfefferbaum 2008). This article will review studies on animal models of alcoholism that indicate multiple mechanisms of alcohol neurodegeneration and loss of key brain functions related to addiction. Other animal studies of brain regeneration in abstinent alcohol-treated animals will be related to human studies investigating changes in the abstinent alcoholic human brain. The integration of animal experimentation with human clinical discoveries supports the significant role of alcohol abuse and abstinence following chronic alcohol abuse in changing brain structure that corresponds with changes in cognition.

## Advantages of Using Animal Models To Study Alcoholic Neurodegeneration

Animal models can be used to clearly test hypotheses about disease factors found in humans. Humans vary in size, weight, age, genetics, diet, and behaviors, including alcohol and tobacco consumption as well as vitamin and aspirin use, exercise, and multiple environmental factors. All of these factors influence health in complex ways that are difficult to untangle when studying people. High-risk alcohol-drinking patterns, including binge drinking (i.e., five drinks for men or four drinks for women in 2 hours) and heavy drinking (i.e., five or more drinks per day for men and four or more drinks per day for women), increase health risks including risk for alcoholism. Alcoholism is a medically defined complex disease with multiple symptoms, the most prominent of which are impulsive and compulsive use of alcohol despite knowing it interferes with mental, physical, and social well-being. Common markers of alcoholism include tolerance to alcohol (i.e., the ability to drink increasingly large amounts) and withdrawal from alcohol (i.e., experiencing bad feelings, tremor, and other symptoms when not drinking). Animal studies of binge and heavy drinking, alcohol tolerance and physical dependence, and the biological effects of alcohol offer the advantage of closely controlling factors that cannot be controlled in human studies. These studies allow researchers to better understand the effects of alcohol on physical and mental health.

Human studies have found reduced gray and white matter in the brains of alcoholics compared with nonalcoholics (see Rosenbloom and Pfefferbaum in this issue, pp. 362–376). Interpreting how much of the reduction in brain size is caused by alcohol consumption may be complicated by variations in brain size among individuals and changes in brain size with age. Human brain volumes decrease with age, and this must be considered when studying the effects of alcohol on the brain (Sullivan and Pfefferbaum 2007). Patterns of drinking vary between people and over time in the same individual, complicating the study of the effect of alcohol on neurodegeneration across individuals (Rosenbloom and Pfefferbaum in this issue. pp. 362–376). Animal models can control for differences in age, quantity and frequency of drinking, diet, genetics, and other factors to understand if alcohol can cause brain damage, how that can change behavior, and what mechanisms underlie alcohol-induced brain damage (Crews et al. 2005). Thus, many factors that complicate human studies can be controlled in animal studies.

## Alcoholic Neurodegeneration: Human and Animal Research Findings

**Figure 1:**
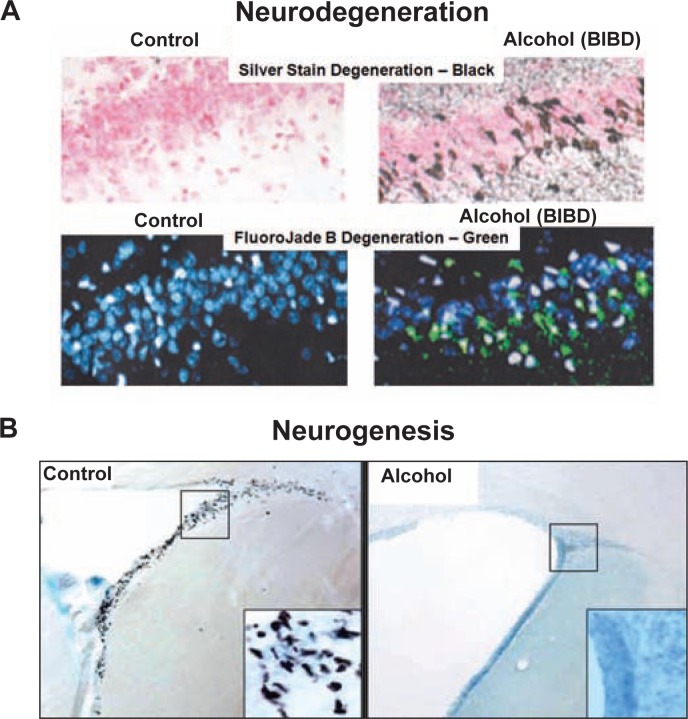
Alcohol-induced neuronal death and inhibition of neurogenesis. **A and B)** Examples of two forms of alcohol-induced neurodegeneration. **A)** Two neuronal cell death stains from controls and the binge-drinking model known as binge alcohol–induced brain damage (BIBD), a 4-day high blood alcohol model with alcohol tolerance and dependence. These panels show sections of the dentate gyrus of the hippocampus from control (left) and BIBD-treated (right) rats. Two neurodegenerative stains, silver stain and fluoroJade B, identify dying neurons, whereas counterstains show all cells. The upper two panels illustrate neurodegeneration silver stain. Pink counterstained cells in controls (left) show no black silver stain cell death, whereas after BIBD (right) neurodegenerative agyrophilic silver stain is prominent (black is positively stained dying cells). FluoroJade B stains dying neurons green. Note that no green cells in controls show blue counterstain. In contrast, BIBD-treated rats have many green dying neurons (right) (for details, see Obernier et al. 2002*a*). **B)** Two images illustrating alcohol inhibition of neurogenesis. Brain sections of frontal cortical subventricular zone are shown. Neural stem cells (NSCs), which differentiate into neurons, appear as black dots (for details see Crews et al. 2006). Note the many black NSCs in the control brain. The inset squares show higher magnification of the stained NSCs. The alcohol-treated animal (acute alcohol 5 gm/kg [right]) shows the effects of alcohol treatment. Alcohol has completely eliminated the NSCs.

**Figure 2:**
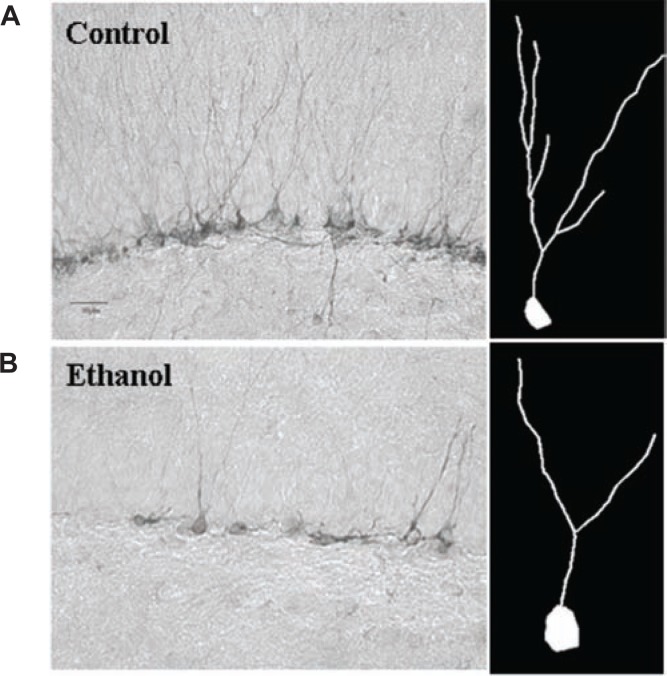
Alcohol reduces new neuron dendritic growth. Neural stem cells (NSCs) in the hippocampus progress from dividing progenitors that exit the cell cycle to grow and differentiate into neurons that are synaptically linked and become fully functional integrated neurons. Doublecortin is a structural protein only expressed in neuronal progenitors differentiating into neurons. Immunohistochemistry for doublecortin labels both the cell body and dendrites of new immature neurons, providing an index of neurogenesis as well as allowing analysis of the effects of alcohol on nerve cell growth. **A, left)** Control immunohistochemistry for doublecortin in the hippocampus. Note that the control has a band of new neuron cell bodies across the section with dendrites extending up from the cell bodies. **B, left)** Alcohol-treated animal doublecortin staining. Note the decreased number and density of new neurons in alcohol-treated animals. **Right:** Representative dendritic trees traced from control (A) or alcohol-treated (B) animals using doublecortin histochemistry. Note how alcohol reduced both the number of new neurons and the size of the dendritic tree showing reduced dendritic length and branching (He et al. 2005).

**Figure 3:**
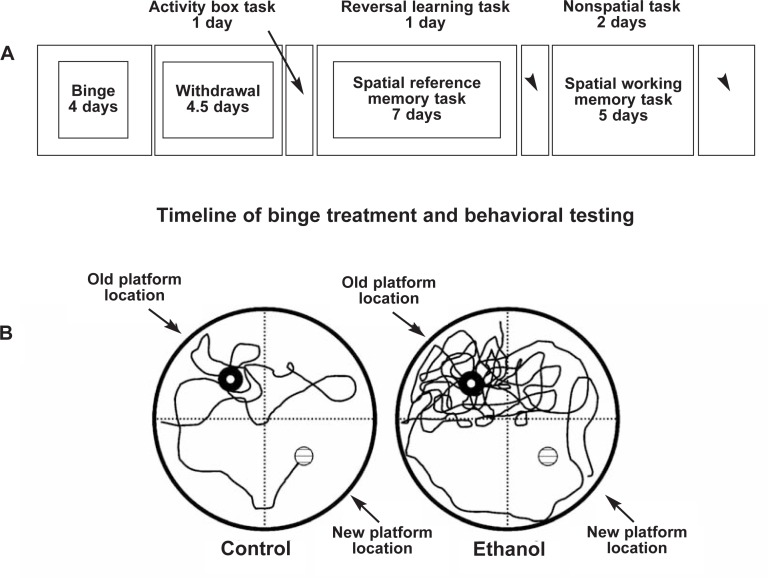
Alcohol-induced persevering, compulsive relearning deficits mimic alcoholic cognitive dysfunction. Shown are the time lines of treatment and testing (upper box) as well as the behavior of two individual rats (bottom two circles), a control (left) and a binge alcohol–treated (right) rat. As indicated in the upper box time line, the 4-day binge alcohol treatment was followed by a 4.5-day abstinent withdrawal period. This model induces physical dependence that can include seizures during withdrawal. Physical withdrawal symptoms subside within 24 hours in this model. It was reasoned that long-term, perhaps permanent, changes in behavior attributed to binge-induced brain damage would be apparent in abstinence beyond withdrawal. After 5 days of abstinence, special reference memory tasks were tested for 1 week using the Morris water maze. The Morris water maze involves learning the location of a hidden platform just under the water using visual cues on the walls surrounding a round water tank 6 feet in diameter. Both control and binge alcohol–treated animals could swim equally well, had normal activity, and easily learned to find the platform. Learning (decreased time to find the platform) with repeated trials was not altered. There was no indication of a persistent binge alcohol treatment effect on learning. However, when relearning tasks were tested at almost 2 weeks of abstinence, a persistent cognitive change was found. Both control and abstinent binge-treated rats readily learned the old platform location. However, reversal learning was disrupted in binge-treated rats. Reversal learning was tested by moving the submerged platform from the original position in the water tank to a position in the quadrant opposite that in which it had been placed during the learning memory task (moved from northeast to southwest quadrant). A vertical view of the tracks taken by control (left) and alcohol (right)-treated rats during the reversal learning task is traced in the two circles representing the water tank. Note the path of the control rat. It first investigates the old learned platform location, reflects on the platform not being in the old location, and then searches and finds the new platform location. Note the persevering of circling behavior shown by the binge-treated animal with numerous reentries into the original goal quadrant. The binge-treated rat failed to reach the new platform location within the maximum time allowed and was removed. Thus, the trace ends in the water and not on the platform. The loss of executive function in the binge alcohol–treated rat is apparent in the repeated, compulsive searching in the old learned position and the lack of cognitive flexibility to search the other areas. SOURCE: Adapted from Obernier et al. 2002*b*.

Human studies (Harper and Kril 1990; Harper and Matsumoto 2005) using brain imaging or examining brains after death have found that alcoholics have smaller brains, particularly frontal cortical regions and white-matter brain regions that represent the wiring connecting the brain. In addition, alcoholics have larger fluid-filled areas (i.e., ventricles) of the brain and smaller overall brain matter. Functional deficits in alcoholics can relate to brain regional size deficits (see Rosenbloom and Pfefferbaum in this issue, pp. 362–376). Animal studies (Crews and Nixon 2008) indicate that alcohol can cause brain damage during intoxication (see figures 1 and 2). Further, alcohol-induced neurodegeneration in animals causes behavioral changes consistent with dysfunctional behavior found in human alcoholics (see figure 3). These studies suggest that alcoholic neurodegeneration could contribute to alcoholism.

Alcoholism is related to genetic and environmental factors that converge to cause this mental disease. Research with animal models of increased genetic risk for alcoholism using the rat model bred for heavy alcohol drinking (i.e., the P alcohol-preferring rat) has found that increased risk for alcoholic brain damage corresponds with increased genetic risk for alcoholism (Crews and Braun 2003). Similarly, studies (Crews et al. 2005) have found that adolescent human drinking increases the lifetime risk for alcoholism, and, in animal models, adolescents show increased alcohol-induced brain damage, particularly in frontal brain regions, consistent with increased biological risk overlapping with increased brain damage. These studies suggest that some of the genetic and biological risk factors for alcoholism are risk factors for alcohol-induced brain damage. It is possible that alcohol and heavy drinking are environmental factors causing degeneration of key controlling brain structures that lead to the compulsive dysfunctional behavior of alcoholism (Crews et al. 2005). The interplay of knowledge from human clinical observation and experimentation with animal models ultimately serves to increase understanding of the elements that cause alcoholism and improve health through advances in prevention and treatment.

## Animal Models of Alcoholic Brain Damage

Animal and human brains are remarkably similar. Animal models therefore allow important detailed investigations of alcohol-induced changes in brain chemistry; gene expression; cellular physiology, including cell proliferation and cell death; as well as the resulting alterations in neuroanatomy and brain function. Alcohol-induced brain atrophy and neurodegeneration is modeled in rats and mice by investigator-administered alcohol, by providing liquid diets that contain alcohol and are the only source of food or by a vapor chamber, all of which achieve high blood alcohol concentrations (BACs). In most cases, complete vitamin-enriched diets are used to assure that alcohol is the damaging agent and not vitamin deficiency. In humans, vitamin deficiencies can cause neurodegeneration. Although most alcoholics do not show pronounced vitamin deficiencies, it is possible that human alcoholic neurodegeneration includes a component related to transient vitamin deficiencies during heavy-drinking episodes (Bowden et al. 2001). Walker and colleagues (1980) were the first to show alcohol-induced neurodegeneration in rats. The study found that feeding rats a nutritious liquid diet containing alcohol for 5 months, followed by 2 months of abstinence, resulted in a loss of brain neurons, specifically types of neurons known as hippocampal pyramidal and dentate granule cells.

### Binge-Drinking Model

Following the initial study by Walker and colleagues (1980), researchers often have used a 4-day investigator-administered binge-drinking model with alcohol beverage solutions painlessly applied down the throat into the stomach (Crews et al. 2004). This is referred to as the “binge-induced brain damage” (BIBD) model (Crews and Nixon 2008). Other methods to model alcohol intoxication include a gas-vapor alcohol treatment to administer alcohol (Zahr et al. 2008) and months of a nutritious liquid diet containing alcohol (Pascual et al. 2007). Research using all of these methods has found evidence of alcohol-induced brain damage, and the models consistently show that brain damage primarily occurs with high BACs. Human hospital admission reports indicate that 4 to 10 percent of emergency-room patients have a BAC over 0.25 percent (Crews and Braun 2003; Crews et al. 2004). These BAC levels have been found to cause neurodegeneration in animal models.

#### Evidence for Alcoholic Neuro-degeneration

Studies of the BIBD model indicate that these BACs can lead to alcoholic tolerance, physical dependence, and at least two forms of neurodegeneration (see figure 1) (Crews and Nixon 2008). Rat models using a vapor method to achieve high BACs have found that weeks of exposure to alcohol lead to ventricular expansion (Pfefferbaum et al. 2008) and alterations in brain chemistry consistent with degeneration (Zahr et al. 2008) in rats, mimicking the neurodegeneration found in human alcoholics. Research with rat and other animal models indicate that high BACs result in neuronal death in multiple limbic cortical brain regions as well as the entorhinal cortex, which relays information to and from the hippocampus and the dentate gyrus, a part of the hippocampus (see figure 1) (Crews et al. 2004). Neurodegeneration, particularly dark-cell degeneration, a necrotic form of cell death marked by shrinking of the cell body (i.e., soma) (Obernier et al. 2002*a*), occurs during intoxication. During alcohol intoxication, markers of neuronal death increase progressively, with multiple brain regions showing increasing damage the more time is spent intoxicated.

A second form of degeneration involves the loss of stem-like cells that are progenitors for the creation of new neurons (i.e., neurogenesis) and cell genesis, such as neural stem cells (NSCs). NSCs form new neurons and nonneuronal support cells (i.e., glial cells) that contribute to brain function and plasticity, particularly mood and memories of complex associations (Crews and Nixon 2008). Alcohol reduces brain NSCs (see figure 1). Neurogenesis occurs throughout life, although levels of NSCs decline with increasing age. Alcohol intoxication inhibits neurogenesis. Adolescent rats have high levels of neurogenesis, but this process is inhibited by low BACs, with high BACs completely destroying neuroprogenitors (see figure 1) (Crews et al. 2006). Alcohol reduces hippocampal neurogenesis by inhibiting NSC proliferation and survival (Nixon and Crews 2002) as well as increasing NSC cell death and blunting the growth of new neurons (see figure 2) (He et al. 2005). As shown in figure 2, alcohol reduces neuron size, which may be indicative of brain-cell shrinkage during alcohol intoxication. Thus, animal studies of alcohol consumption have found neurodegeneration through nerve-cell death as well as cell shrinkage, as evidenced by the small dendritic trees in new neurons exposed to alcohol and the inhibition of ongoing cell genesis and neurogenesis (see figures 1 and 2). These findings suggest that human alcoholic neurodegeneration likely is a composite of neuronal death, inhibited creation of new neurons, and shrinkage of existing neurons and glia.

#### Effects of Neurodegeneration on Brain Function

The finding that chronic alcohol in rat models causes diffuse neurodegeneration across multiple brain regions, similar to that found in human alcoholics, is consistent with evidence that alcohol causes human neurodegeneration. Although alcoholic neurodegeneration is diffuse, both human and animal studies have suggested that the front parts of the brain (i.e., the frontal and prefrontal cortex) are particularly sensitive to this type of damage. Brain frontal cortical regions are responsible for attention, impulse inhibition, and as reflective decision processing.

People with frontal cortical lesions show a loss of a reflective analysis of future pain or pleasure and increased anxiety and negative mood, which contributes to a loss of impulse control (Bechara 2005). This is similar to the impulsive–compulsivity of alcoholism and other addictions. Lesions in the frontal cortex also disrupt relearning, likely because of a loss of assessment of searching strategy, mimicking some aspects of addiction (Schoenbaum and Shaham 2008).

Human alcoholic studies are complicated by a lack of knowledge of brain size and function before alcoholism. Studies in rats have found that weeks after binge alcohol–induced brain damage, behavioral tests show relearning deficits compared with controls (see figure 3) (Obernier et al. 2002*b*). These findings suggest that high BACs alter brain structure and behavior in a way that mimics the behavioral dysfunction of alcoholism.

A narrowing of activities with increasing repeated behaviors is a symptom of human alcohol use disorders. The subtle cognitive deficits associated with the “processing inefficiency” found in human alcoholics are varied and difficult to model in animals. However, the rat studies finding both neurodegeneration and increased impulsive–compulsive behaviors similar to alcoholic human behavioral dysfunction suggest that these dysfunctions are at least in part caused by heavy drinking and high BACs.

Although human studies find that alcoholic cognitive deficits correlate with brain region size deficits, these could have preexisted and contributed to the development of alcoholism (Rosenbloom and Pfefferbaum in this issue, pp. 362–376). Animal studies suggest that heavy drinking contributes to the changes in alcoholic behavior and brain structure. Further, rat studies show that genetics (Crews and Braun 2003) and adolescent age (Crews et al. 2000) are risk factors for alcohol-induced neurodegeneration. Human studies (Crews et al. 2005) show that genetics and adolescent drinking are risk factors for human alcoholism, consistent with alcohol-induced neurodegeneration altering cognition, increasing risk, and possibly causing alcoholism. Thus, human alcoholism and human alcoholic neurodegeneration and cognitive dysfunction can be explained by heavy alcohol drinking and are not necessarily innate in the alcoholic human brain. The following section will examine the processes that may contribute to alcohol-related neurodegeneration.

## Mechanisms of Alcohol-Related Neurodegeneration

**Figure 4:**
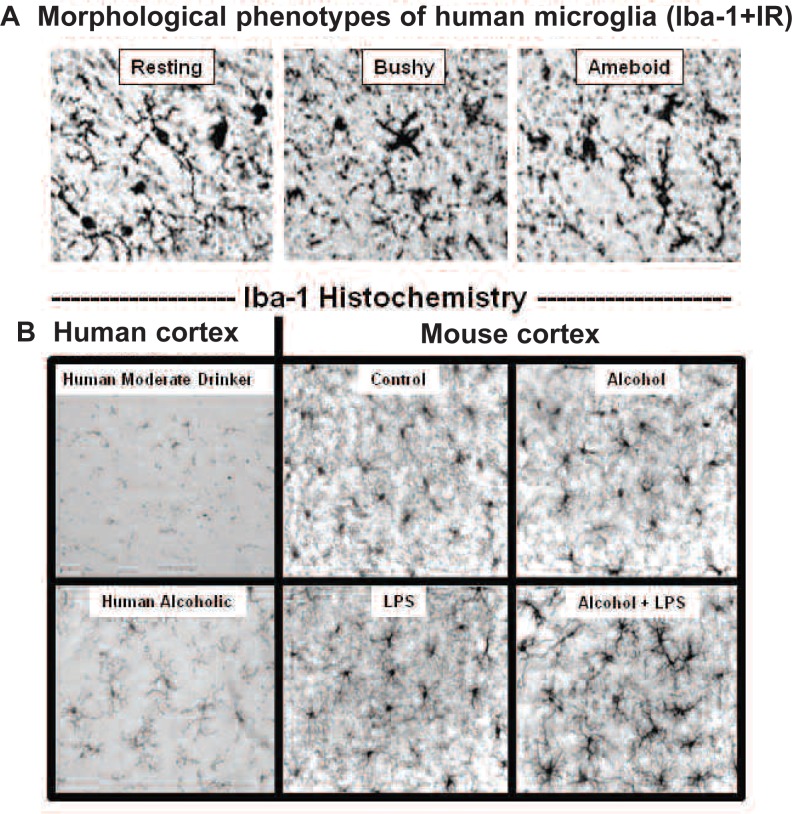
Microglial markers in human and mouse alcoholic brain. **A)** Images of human microglia. Microglia take on different shapes depending upon the cellular state (e.g. healing or inflammatory). Shown are images of human microglia in multiple states or stages of activation, identified using Iba-1+immunoreactivity (IR). Resting, healing microglia have many small sensing arms that can secrete trophic factors and strengthen neurons (top left). Activated microglia start to produce large amounts of proinflammatory cytokines, cyclo-oxygenase, and NADPH oxidase. Their arms and bodies thicken making them bushy (top middle). Ameboid microglia (top right) are phagocytizing cells (for details, see He and Crews 2008). **B)** Human alcoholics have increased microglia in the cortex. Iba1+IR is greater in postmortem alcoholic brain compared with moderate drinkers (panels on left side). Studies in mice have found that alcohol, particularly alcohol with bacterial endotoxin (lipopolysaccharide, LPS), increased the density and morphology of microglia (see four panels on right side) (Qin et al. 2008).

The mechanisms of alcohol-induced neurodegeneration are complex and share many processes with other neurodegenerative conditions. Chronic alcohol consumption leads to increased activation of glial cells known as astrocytes and microglia^1^ (see figure 4) as well as increased expression of brain proinflammatory genes, all indicators of neurodegeneration and brain damage.

### Timing of Alcohol-Induced Brain Damage

Human studies showing mild alcoholic neurodegeneration and reduced cognitive ability cannot determine when the deficits occurred. That is, did alcoholics have these deficits before starting drinking, induce them during drinking, or induce them during withdrawal from repeated drinking–abstinence–withdrawal episodes? Animal studies indicate that alcohol-induced brain damage occurs largely during intoxication, requires relatively high BACs, and occurs in the absence of marked nutritional deficiency, seizures, or other brain injuries. Multiple histological techniques have been used to show neuronal cell death in animals. Time course studies of neuronal death are complicated by a long delay, often days, between the fatal triggering of neuronal death and detection of dying neurons and glial activation markers in brain. However, several studies using multiple markers of neurodegeneration at various times during chronic alcohol intoxication (Obernier et al. 2002*a*,*b*) and during alcohol withdrawal and periods of abstinence following the alcohol withdrawal syndrome allow a determination of when neurons die (Crews et al. 2000, 2004) and when neurogenesis is inhibited (Crews et al. 2006; He et al. 2005). These time course studies indicate that alcoholic degeneration increases during intoxication at high BAC and progressively subsides during abstinence (Crews and Nixon 2008). Although markers of neurodegeneration decline in abstinence, glial activation and proinflammatory gene expression may persist for long periods in the brain, suggesting that repeated high BACs would worsen neurodegeneration.

Human studies are consistent with neurodegeneration during intoxication, with recent and frequent heavy drinking being the best indictor of alcoholic brain damage (Parsons and Stevens 1986; Sullivan and Pfefferbaum 2005). Frontal cortical metabolites, specifically choline-containing compounds measured by magnetic resonance imaging (MRI), are increased in brain damage and in alcoholics with significant correlations between alcohol consumption in the last 90 days and increases in frontal cortex (Ende et al. 2006), suggesting that damage correlates with recent alcohol consumption. Similarly, studies in rats have found increases in brain choline-containing compounds during chronic alcohol treatment with increased time intoxicated and higher BACs, further increasing choline compounds in the brain (Zahr et al. 2008). Thus, high BACs best predict human and animal alcoholic neurodegeneration.

### Microglia

Microglia are brain immune defense cells, and they constitute about 20 percent of the cells in the brain but can proliferate when activated. Activated microglia can express many proinflammatory genes, including cyclo-oxygenase, an enzyme that is inhibited by aspirin, ibuprofen, and other anti-inflammatory drugs (Knapp and Crews 1999). NADPH oxidase, a membrane-bound enzyme complex, is proinflammatory, producing activated oxygen free radicals that can burn bacteria and other cells. Proinflammatory genes also code for a group of signaling hormones called cytokines that can change immune and nerve cell function. Microglia can support neurons and have healing functions or can become activated to induce large amounts of proinflammatory proteins.

Microglia take on different structures (phenotypes) during different activities. Resting, healing microglia have many small sensing arms (see figure 4, *top left*). Activated microglia start to produce large amounts of proinflammatory cytokines, such as tumor necrosis factor α (TNF-α) and other proinflammatory genes, including cyclo-oxygenase and NADPH oxidase. When activated, their arms and bodies thicken, making them bushy (see figure 4, *top middle*). Ameboid microglia (see figure 4, *top right*) are phagocytizing cells that clear infectious agents, cellular debris, and toxic agents and are associated with extensive degeneration as is found in stroke and brain trauma. In normal responses to wounds, invading organisms are killed by ameboid cells, until a signal to the activated ameboid cells indicates that the invasion is over and the cells stop killing and change to healing. A great deal of research is underway on these signals. In human and animal brains, morphological phenotypes of microglia become diverse in upper-middle and advanced age, complicating the understanding of microglial function after middle age.

Similar to many human neurodegenerative diseases, postmortem alcoholic human brains have increased microglial-specific cellular markers compared with age-matched control subjects (see figure 4, *bottom left*). Alcoholic human brain also has more proinflammatory cytokines (He and Crews 2008). Treating mice with binge-drinking amounts of alcohol and/or lipopolysaccharide (LPS) (a bacterial component in the gut that leaks into the body during alcohol drinking) results in changes in microglial morphology that mimic those found in human alcoholic brain tissue (see figure 4, *bottom right*). Microglia are likely to contribute to alcoholic neurodegeneration, although their role still is under investigation and other cells clearly contribute. These findings indicate that the changes in alcoholic human brain are related to heavy alcohol drinking and possibly a leaky gut, because alcohol tends to allow bacterial endotoxin to leak from the gastrointestinal tract into the blood, which increases blood proinflammatory cytokines. These cytokines then are transported into the brain and activate brain proinflammatory gene induction (Qin et al. 2007, 2008). The neurodegeneration in alcoholism therefore appears to be related, at least in part, to changes in microglia and proinflammatory gene expression.

### Proinflammatory Genes

There are at least two mechanisms of alcohol-induced brain proinflammatory gene induction, and both require high BACs (Crews and Nixon 2008). One involves leakage of endotoxin from the gut and resulting increases in proinflammatory cytokins, as described above. In addition, alcohol directly increases transcription of brain proinflammatory genes. Genes are encoded in DNA, the genetic material. A transcription factor (sometimes called a sequence-specific DNA binding factor) is a protein that binds to specific sequences of DNA in a gene or groups of genes (proinflammatory genes in this case) and thereby controls the conversion (i.e., transcription) of the gene from DNA to RNA. RNA then is translated into proteins, which make up the enzymes, cell-structural skeletons, cytokines, and many other components of brain cells. Regulation of gene transcription is complicated and involves many processes. However, studies have gained insight by examining the binding of protein transcription factors to specific sequences of DNA. Two important transcription factors altered by alcohol are cAMP-responsive element-binding protein (CREB) and a transcription factor first discovered in activated B lymphocytes, nuclear factor κB (NF-κB) (Zou and Crews 2006). These transcription factors bind to different specific gene–DNA sites and thereby regulate how much protein is made from those genes. Alcohol increases DNA binding of NF-κB and decreases DNA binding of CREB in association with increases in the transcription of proinflammatory genes, such as cytokines and inflammatory enzymes, and decreases in amounts of CREB-induced growth factor protein (Zou and Crews 2006). These alcohol-induced changes in brain gene transcription overlap with the transcription of genes important for memory and other forms of drug addiction (Lonze and Ginty 2002; Nestler 2002).

***NF-κB.*** NF-κB is a transcription factor widely known for its ubiquitous roles in inflammatory and immune responses and in control of cell division and programmed cell death. NF-κB is activated by alcohol as well as by oxidative stress, cytokines, and the neurotransmitter glutamate (Madrigal et al. 2006). Increased NF-κB has been found in the dying neurons of brains exposed to trauma and reduced blood supply and in patients with Alzheimer’s disease and Parkinson’s disease (Zou and Crews 2006). Activation of NF-κB transcription increases proinflammatory cytokines, with TNF- α being the prototype. Human alcoholic brain shows increased NF-κB gene transcription (Okvist et al. 2007) as well as increased proinflammatory cytokine and microglia protein expression (He and Crews 2008). Similarly, animal studies have found alcohol-induced proinflammatory gene expression with neurodegeneration (Crews et al. 2006; Qin et al. 2008). Human genetic variations in NF-κB genes have been associated with increased risk for alcoholism, particularly early-onset alcoholism (Edenberg et al. 2008). Further, analyses of genes expressed in postmortem human alcoholic brain find large differences in genes related to NF-κB transcription, proinflammatory genes, and other genes associated with neurodegeneration (Liu et al. 2006; Okvist et al. 2007; Liu 2004). Similarly, studies investigating brain gene expression in animals modeling alcoholism find that these groups of genes are altered (Mulligan et al. 2006).

Taken together, these findings suggest that high BACs increase expression of proinflammatory genes in the brain, thereby increasing oxidative stress and triggering glial cell activation that contributes to neuronal death and further promotes proinflammatory gene expression. Interestingly, proinflammatory cytokines found in alcoholic human brain (He and Crews 2008) increase the reward value of alcohol drinking in mice (Blednov et al. 2005). The genes identified as altered in animals that prefer to drink large amounts of alcohol overlap with proinflammatory genes and neurodegeneration (Mulligan et al. 2006). Alcoholic neurodegeneration is prominent in the frontal cortex and likely contributes to impulsive–compulsive alcohol seeking and consumption in the presence of negative consequences, a hallmark of alcoholism.

These studies suggest that high BACs, proinflammatory cytokines, and neurodegeneration may be significant contributors to alcoholism.

## Blocking the Mechanisms of Neurodegeneration

Evidence supporting the role of proinflammatory genes and oxidative stress in alcoholic brain damage is found by studying drugs that block neurodegeneration. Butylated hydroxytoluene (BHT) is an antioxidant that uniquely blocks alcohol-induced increases in DNA binding of NF-κB, proinflammatory gene induction, and alcohol-induced decreased DNA binding of CREB (Zou and Crews 2006). BHT given to rats before and during the BIBD model prevented increased brain NF-κB–DNA binding, proinflammatory gene induction, the loss of neurogenesis, and neurodegeneration (Crews et al. 2006; Hamelink et al. 2005). Similarly, increasing transcription of pCREB, the active form of CREB, through the use of drugs can block neuroinflammation and alcohol-induced brain neuronal death (Zou and Crews 2006).

Some dietary antioxidants and other anti-inflammatory agents may be protective against alcohol-induced brain damage. Thus, genetic and environmental alterations in alcohol-induced proinflammatory gene induction can regulate alcohol-induced inhibition of neurogenesis and neurodegeneration.

## Brain Regeneration During Abstinence

If alcohol-induced changes in brain structure, physiology, and gene expression contribute to the loss of control over drinking, it follows that regaining control during abstinence could involve changes in brain structure, physiology, and gene expression that contribute to the return of control and recovery from addiction (Crews et al. 2005). Alcoholics recognize the cognitive inefficiency that occurs during heavy drinking and call it “a wet brain.” This is surprisingly accurate when thinking about alcoholics’ enlarged brain ventricles, which are spaces in the brain containing cerebral fluid. People in recovery have reported that their cognition improves with the duration of abstinence. Multiple well-controlled studies (Crews et al. 2005) of alcoholics have found evidence that alcoholic sobriety results in improved brain function, metabolism, and volume during abstinence. Increased brain volume corresponds with decreased ventricular size (i.e., less water in the brain) (Sullivan et al. 2000; Volkow et al. 1994). These studies are complicated by the lack of brain measures before abstinence, high rates of relapse, and variability among individuals. In large part, most studies indicate improvement of brain function during abstinence. The longer the abstinence, the greater the chances of maintaining a healthy recovery from addiction and return of executive functions (Crews et al. 2005).

### Neural Stem Cells (NSCs)

Adult NSCs self-renew and differentiate into all types of neural cells (Zhao et al. 2008). NSC proliferation and differentiation is sensitive to experiences, activities, physiology, and drugs. NSCs proliferate, migrate, differentiate, and integrate into existing brain circuits that contribute to learning complex associations (Zhao et al. 2008). Although NSCs are present throughout human and mammalian brains, hippocampal and frontal brain regions have highly active NSCs, forming many new neurons daily into old age (Crews and Nixon 2003; Taupin and Gage 2002). The formation of new neurons takes time. It takes months for NSCs to progress from proliferation, migration, and differentiation of dendritic and axonal connections, to appropriate integration into existing brain circuits (Zhao et al. 2008). NSCs respond to the environment throughout this process. The number of new neurons in the adult hippocampus is reduced by stress, alcohol, and cytokine–proinflammatory gene expression. Learning, exercise, antidepressant treatment, and withdrawal from alcohol dependence increase new hippocampal dentate gyrus neurons. Increasing neurogenesis increases learning and mood, whereas decreasing neurogenesis appears to disrupt learning and mood. These changes in neurogenesis likely reflect brain plasticity. Learning, memory, and other forms of plasticity likely are attributed to changes in brain circuitry, with neurogenesis representing a mechanism of altering circuitry.

### Abstinence Following Binge Drinking

**Figure 5:**
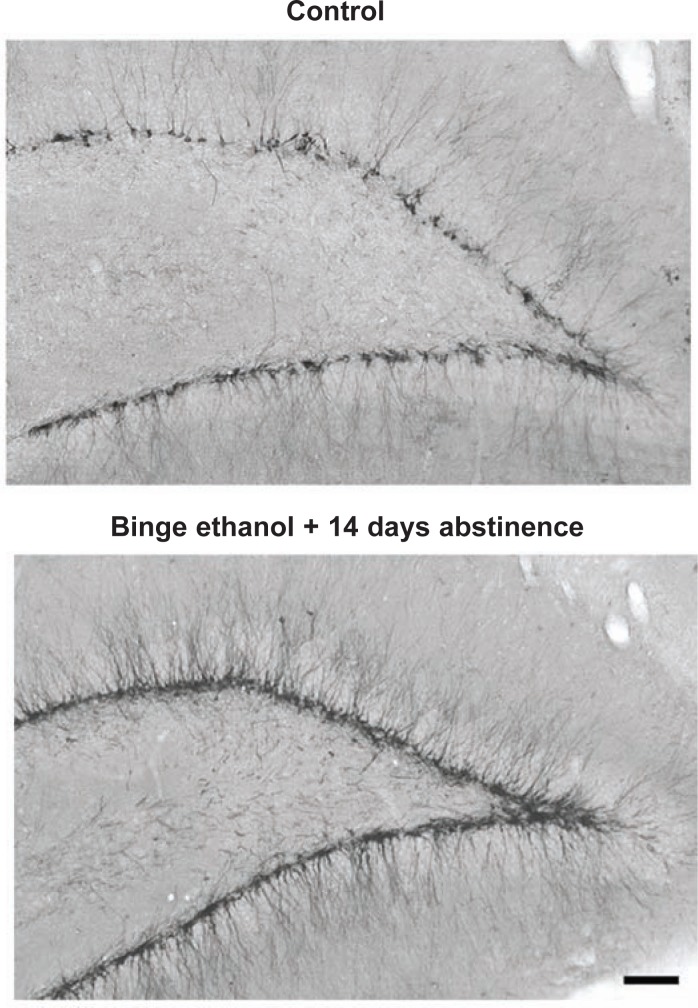
Neurogenesis during abstinence following binge alcohol treatment. The protein doublecortin (DCX) is expressed in neuroprogenitors during differentiation into mature neurons (Brown et al. 2003). The images show DCX immunohistochemistry in control subjects and after 14 days of abstinence following a 4-day binge-drinking period. Note the prominent increase in new neurons (exhibited by dark staining) being formed after 2 weeks of abstinence (Nixon and Crews 2004).

**Figure 6:**
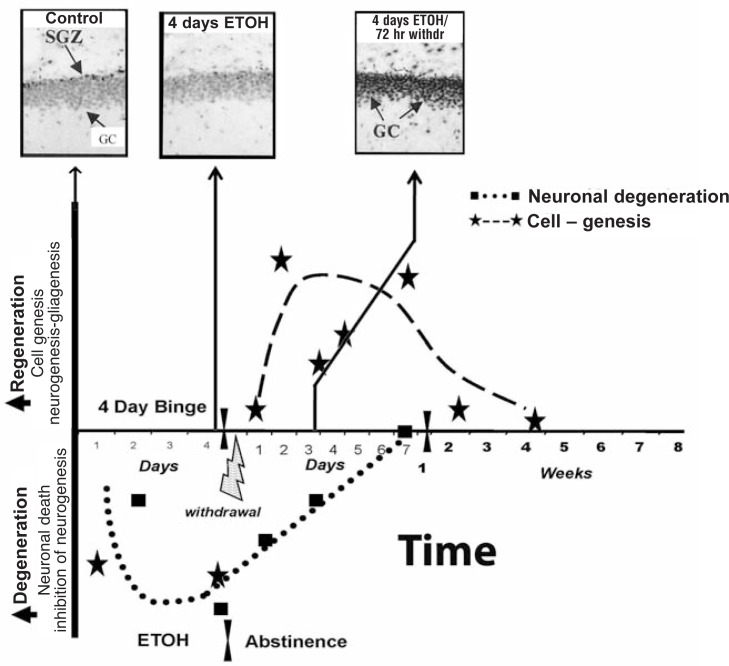
Regeneration of brain is related to increased phosphorylated cAMP-responsive element-binding protein (pCREB). The 4-day rat BIBD model time line illustrates the relationship between alcohol-induced degeneration, abstinence-induced neurogenesis, and pCREB. The temporal relationship of binge-induced neurodegeneration and abstinence-induced cell genesis can be examined by pCREB immunohistochemical staining in the dentate gyrus. Immunohistochemical staining is a process of localizing proteins in cells of a tissue section using antibodies that bind to specific proteins, such as pCREB. More staining means more protein. In the dentate gyrus granule cells (GCs) of control subjects, most neuronal nuclei have some pCREB+ immunoreactivity (IR), with higher levels of staining in the subgranule zone (SGZ), where neurogenesis is active (control, upper left image). In the diagram, values below the x-axis reflect degeneration or loss of brain mass. Markers of neuronal death increase throughout the 4 days of intoxication. Neurogenesis decreases and pCREB+IR is low (middle image). Markers of neuronal death persist into abstinence, although they progressively decline and mostly disappear after 1 week of abstinence (dotted line). Regeneration is represented by the dashed line increasing above the x-axis, with stars indicating time points of measured neurogenesis and other cell genesis (Crews and Nixon 2008). After 4 days of binge alcohol treatment, pCREB staining is decreased when neurogenesis is inhibited and granule cells degenerate. However, after 72 hours of abstinence, a marked increase in pCREB staining (top photo [4 days alcohol/72 hours withdrawal]) coincides with increased neurogenesis and loss of degeneration markers (Bison and Crews 2003). NOTE: CREB is a transcription factor altered by alcohol. When CREB is activated, pCREB is formed. The dentate gyrus is part of the hippocampus.

Animal binge-drinking models investigating NSCs and neurogenesis have found that alcohol inhibits neurogenesis, with adolescents being particularly sensitive (Crews and Nixon 2008). Abstinence following binge-drinking treatment in rats results in increased NSC proliferation in multiple brain regions (Nixon and Crews 2004). NSC proliferation increases within 1 day of abstinence and continues for many days and weeks (He et al. 2008). During abstinence following alcohol dependence, hippocampal NSCs proliferate in bursts, with an expansion of cells leading to a progressive wave of cells differentiating from NSCs into immature new neurons. Specific proteins, such as doublecortin, are expressed only in developing new neurons. This allows researchers to use this protein to identify new neurons. As shown in figure 5, hippocampal neurogenesis is dramatically greater in rats 2 weeks after the last dose of alcohol in the 4-day binge model compared with age-matched controls not exposed to alcohol. The alcohol-abstinence–induced burst of cell proliferation occurs as the degeneration and fragments of dying neurons clear but also is associated with a marked increase in pCREB, likely caused by synaptic glutamate-activating trophic signals. Trophic, cell-strengthening signals increase through pCREB, as described in figure 6. Multiple broad areas of the brain show new progenitor cells at 1 and 2 months of abstinence. New cells in the hippocampus become neurons, whereas in many other brain regions they become microglia but do not appear to be activated proliferating microglia (Crews and Nixon 2008). Although microglial proliferation is a sign of proinflammatory microglial activation, the lack of a “bushy” or “ameboid” morphology suggests that proliferating progenitor cells become resting microglia. Although studies of humans have limited information on lifetime cycles of drinking and abstinence, the increase in microglia in abstinent rat brain is similar to the increased numbers of microglia found in human alcoholic brains (see figure 4).

More recent studies of abstinence following alcohol self-administration in rats found increased NG2 neuroprogenitors. NG2 is a marker of progenitors that often become oligodendrocytes, the cells that make myelin, the insulation of brain circuits (He et al. 2008). This could possibly indicate the regrowth of glial cells, particularly oligodendrocytes, with abstinence. In longitudinal human studies of recently abstinent alcoholics, volumetric brain gain during abstinence was found to be related to metabolic and neuropsychological recovery and increased cerebral choline, consistent with glial growth and remyelination contributing to abstinent alcoholic brain growth (Bartsch et al. 2007). Cell genesis during abstinence is brain growth. Genesis of neural stem cells, microglia, oligodendrocytes, astrocytes, and neurons during alcohol abstinence represents a unique and long-term change in brain-cell structure that persists for long periods, perhaps permanently. These findings suggest that humans who vary their alcohol consumption over periods of time are undergoing continuous degenerative and regenerative cycles that follow the drinking and abstinence cycles. Therapies that enhance abstinence-induced brain regrowth may be useful in drug dependence and other mental diseases.

## Mechanisms of Abstinence-Increased Cell Genesis and Brain Growth

The mechanisms of abstinence-induced increases in neurogenesis are not known. One likely factor is that proinflammatory gene expression declines in the absence of alcohol but continues at a lower level. Abstinence from alcohol also involves a transient withdrawal hyperexcitability associated with increased synaptic glutamate and other transmitter release (De Witte et al. 2003). Although excessive glutamate is associated with neurotoxicity, synaptic glutamate release is associated with increased activation of CREB (i.e., pCREB formation), as well as increased synthesis and secretion of trophic factors (Zou and Crews 2006). Alcohol increases excitatory synapse size, which could increase trophic synaptic glutamate responses during abstinence (Carpenter-Hyland et al. 2004). In rats, 4-day binge drinking–induced neurodegeneration and loss of neurogenesis corresponds with decreased hippocampal pCREB immunohistochemistry (see figure 6, *top middle picture*). The pCREB that was decreased during high BAC, when neuronal degeneration occurs (see figure 6, *squares*), reverses and dramatically increases during abstinence (see figure 6, *top right*). This increase in CREB activation during abstinence corresponds with the time course of increased neurogenesis (see figure 6, *stars above midline*). This increase in pCREB could increase plasticity, cell growth, cell proliferation, and neuroge-nesis. Thus, regeneration during alcohol abstinence likely involves increased trophic signaling and reduced proin-flammatory gene expression, which contributes to progenitor cell genesis and possibly additional trophic brain growth responses.

## Summary

Animal studies have established that high BACs can cause neurodegeneration similar to that found in human alcoholics. The rat BIBD model causes neurodegeneration in multiple brain regions directly related to alcohol rather than diet or alcohol withdrawal. Neuronal cell death, as well as the inhibition of neurogenesis, contributes to alcohol-induced brain degeneration. Models of binge alcohol consumption in rats produce changes in cognition similar to the executive function processing inefficiencies found in human alcoholics. Risk factors for alcoholism overlap with risk for alcoholic brain damage. The mechanisms of brain damage appear to involve proinflam-matory cytokines, oxidative stress, and loss of trophic factors—mechanisms that overlap with many comorbid mental and neurodegenerative diseases. Abstinence following alcohol dependence results in neural stem-cell proliferation and the formation of new neurons and other brain cells indicating brain growth. These findings provide insight into when, where, and how alcohol abuse and abstinence–recovery dynamically change brain-cell composition, which could lead to new potential therapies for neurodegeneration, mental diseases, and alcohol use disorders.

## Footnotes

1 The definition for this ad other technical terms can be found in the Glossary, pp. 345–347.
